# ASO Author Reflections: Defining the Role of PIPAC in Ovarian Cancer – Results from a U.S. Phase 1 Clinical Trial

**DOI:** 10.1245/s10434-025-18588-9

**Published:** 2025-10-22

**Authors:** Brad Nakamura, Tri A. Dinh, Thanh H. Dellinger

**Affiliations:** 1 Department of Surgery, City of Hope National Medical Center, Duarte, CA USA; 2 Department of Medical and Surgical Gynecology, Mayo Clinic, Jacksonville, FL USA

## PAST

Ovarian cancer (OC) often presents with peritoneal metastases (PM), which contribute significantly to morbidity and treatment resistance. Intraperitoneal (IP) chemotherapy combined with optimal cytoreductive surgery improves survival outcomes, both as normothermic and hyperthermic treatment. While hyperthermic intraperitoneal chemotherapy (HIPEC) is associated with overall survival benefit in patients with OC, applicability is largely limited to the first-line setting, highlighting the need for localized therapies in the recurrent patient population. Pressurized intraperitoneal aerosol chemotherapy (PIPAC) has emerged as a promising modality for locoregional drug delivery in peritoneal surface malignancies, including in OC. Several single-arm phase II trials in Europe have demonstrated encouraging responses in patients with OC.^1,2^ These trials, however, enrolled patients with OC with disease limited to the peritoneal cavity, thus omitting a large portion of recurrent patients with OC affected by extraperitoneal and distant metastases. Evaluating efficacy in previous PIPAC trials in OC has also been hampered by inconsistent outcome measures across trials, such as varying laparoscopic scoring and histologic regression outcomes.

## PRESENT

The current study represents the first U.S. trial to establish the safety and feasibility of PIPAC in patients with ovarian cancer across U.S. academic centers.^3^ This phase I trial demonstrates the safety of cisplatin and doxorubicin PIPAC in recurrent patients with OC. In contrast to prior PIPAC studies, this study allowed enrollment of patients with OC with extraperitoneal metastases, which are characteristic of recurrent patients with OC. Generally, a more heavily pretreated OC cohort with multiple prior lines was enrolled. Secondary outcome measures included laparoscopic peritoneal carcinomatosis index (PCI), histologic peritoneal regression grading score (PRGS), and RECIST 1.1 criteria. While not powered to demonstrate efficacy of cisplatin/doxorubicin PIPAC, the study did not demonstrate significant treatment response in a heavily pretreated OC population with extraperitoneal metastases. Radiologic best response (RECIST) was 7%, with one partial (though durable) response, and a stable disease rate of 27%. In contrast, intraperitoneal disease control was significant, with decreased PCI in 31% of patients and histologic regression in 46% of peritoneal tumors, which was comparable to prior PIPAC OC trials.

## FUTURE

The current and prior studies have established safety and feasibility for PIPAC cisplatin/doxorubicin in OC. However, PIPAC monotherapy efficacy may be limited to recurrent patients with OC exclusively with intraperitoneal disease, i.e., without extraperitoneal metastases. Development of future trials that address the typical disease distribution of recurrent patients with OC would expand application of PIPAC to the general recurrent OC population. The future of PIPAC in OC thus lies in the use of multimodal therapy to leverage PIPAC with systemic therapies. Identifying optimal chemotherapies best suited for patients with platinum-resistant recurrent ovarian cancer is essential to integrate PIPAC with modern systemic therapies. In an expansion arm of the current study, we address this gap by incorporating PIPAC nab-paclitaxel with systemic nab-paclitaxel as multimodal therapy to improve the efficacy of PIPAC in the recurrent OC population.^4^ The future thus lies in combining PIPAC with multimodal intravenous (IV) chemotherapy to control extraperitoneal tumor burden, while leveraging clinically active drugs in platinum resistant recurrent OC to accurately evaluate its therapeutic value and real-world applicability.
